# Green Approaches Based on Biodegradable Polymers for Sustainable Electrochemical Sensors

**DOI:** 10.3390/s26154787

**Published:** 2026-07-28

**Authors:** Ece Ozkan, Batuhan Ozturk, Ismail Murat Palabıyık

**Affiliations:** 1 Department of Analytical Chemistry, Faculty of Pharmacy, Ankara Medipol University, 06050 Ankara, Türkiye; batuhan.ozturk@ankaramedipol.edu.tr; 2 Department of Analytical Chemistry, Graduate School of Health Sciences, Ankara University, 06100 Ankara, Türkiye; 3 Department of Analytical Chemistry, Faculty of Pharmacy, Ankara University, 06100 Ankara, Türkiye

**Keywords:** biodegradable polymers, electrochemical sensors, green analytical chemistry, sustainable sensing, chitosan, cellulose, alginate, starch, biosensors, nanocomposites

## Abstract

Electrochemical sensors have become integral components of modern analytical science owing to their exceptional sensitivity, rapid analytical performance, portability, and low operating costs. These advantages have facilitated their broad implementation in environmental monitoring, clinical diagnostics, food safety, and pharmaceutical analysis. However, the increasing use of non-biodegradable materials in sensor fabrication has raised concerns regarding electronic waste and environmental sustainability. This review examines the recent progress in the development of green electrochemical sensors based on biodegradable polymers, with a particular focus on cellulose, chitosan, alginate, and starch. The chemical structures, physicochemical properties, and functional characteristics of these biopolymers are discussed in relation to their roles as sensing matrices and electrode modification materials. Representative applications reported in the literature are systematically reviewed, highlighting their use in the detection of pharmaceuticals, biomolecules, pathogens, heavy metals, pesticides, and environmental pollutants through various analytical techniques, including differential pulse voltammetry, square wave voltammetry, amperometry, electrochemical impedance spectroscopy, fluorescence, and colorimetric methods. The integration of biodegradable polymers with nanomaterials is also evaluated in terms of improving sensor sensitivity, selectivity, and stability. The incorporation of biodegradable polymers into electrochemical sensing platforms offers a sustainable pathway toward next-generation analytical technologies. By balancing high sensing efficiency with a lower environmental footprint, these materials support the transition toward analytical systems designed in accordance with the principles of green chemistry and green analytical chemistry.

## 1. Introduction

Electrochemical sensors have emerged as versatile analytical apparatus for the identification of both chemical and biological analytes largely, and it is the reason that they have been used in the last few decades owing to their great sensitivity, fast response, portability, and low cost [1]. These sensors function by translating chemical or biochemical interactions at the electrode surface into experimental electrical signals such as current, potential, and impedance. Because of the above advantages, electrochemical sensors are widely used in a wide range of applications, among which are environmental monitoring, clinical diagnostics, food safety, pharmaceutical analysis, among others. Compared to analytical devices (like chromatography or mass spectrometry), the electrochemical sensing platform has more straightforward instrumentation, lower energy consumption, possibilities of real-time analysis, and may be miniaturized [2]. Yet, raw materials that are widely used as a source of sensor fabrication can be petroleum-based polymers as well as non-biodegradable substrates, causing environmental pollution once the components have been discarded [3]. More recently, environmental alternatives for developing sensing-based devices are being investigated due to the growing amount of electronic waste accumulation. The growing global demand for sustainable materials has positioned biodegradable polymers at the forefront of scientific research and industrial innovation. Owing to their ability to decompose into environmentally benign products through natural biological processes, these materials have emerged as promising alternatives to conventional synthetic polymers. Despite substantial advances in this field, the development and application of biodegradable polymers remain highly diverse and fragmented, reflecting the wide range of feedstock sources, synthesis strategies, degradation pathways, and application-specific performance requirements [4]. In this light, biodegradable and biocompatible materials have recently attracted considerable interest as potential sustainable materials for next-generation sensors. Biodegradable polymers can decompose into non-toxic products under environmental or physiological conditions, and they could be the most promising solution towards reducing the environmental impact of electronic devices and analytical platforms [5]. Out of all biodegradable polymers, chitosan has garnered significant attention for electrochemical sensing applications. Chitosan is a natural polysaccharide obtained from the deacetylation of chitin and has amino and hydroxyl functional groups. These functional groups enable the immobilization of biomolecules and enhance the interaction between the sensing interface and the target analyte [6]. Chitosan has several desirable properties, including biodegradability, biocompatibility, high adsorption capacity, and film-forming ability, making it a highly favorable platform for the fabrication of electrochemical sensors [7]. Moreover, the combination of biodegradable polymers with nanomaterials and conductive elements has greatly improved the performance of electrochemical sensors. The addition of materials such as carbon nanotubes, graphene, metallic nanoparticles, and conducting polymers can enhance electron transfer, increase surface area and improve the sensitivity and selectivity of the sensing platform. These hybrid systems combine the environmental benefits of biodegradable materials with the high analytical performance required for modern sensing technologies [2].

Alginate hydrogels have an extracellular structure similarity and are an appealing 3D environment for immobilization and biosensor formation of biomolecules. It is well established that all these characteristics render alginate a favorable matrix for electrochemical sensing structures, especially for the realization of eco-friendly or biodegradable sensor systems. Moreover, alginate hydrogels offer high biocompatibility, high water-retention capacity, and mild gelation conditions, thus helping to maintain the biological activity of immobilized enzymes, antibodies, or other biomolecules. Alginate’s porous network structure allows for efficient mass transfer of analytes and reaction products, making electrochemical biosensors more sensitive and faster in response time. Moreover, the physicochemical properties of alginate hydrogels can be readily adjusted by crosslinking density or via incorporation of nanomaterials including metal nanoparticles, carbon nanomaterials, conductive polymers, etc. The tuning of the device greatly impacts its electrical conductivity, mechanical stability, and sensing capability as a whole [8,9].

Cellulose has emerged as one of the most extensively investigated biodegradable materials for electrochemical sensor fabrication owing to its renewable origin, excellent mechanical properties, biodegradability, and outstanding film-forming capability. Its abundant hydroxyl functionalities provide versatile sites for chemical modification and the immobilization of sensing elements, while nanocellulose derivatives offer a high specific surface area that facilitates analyte accumulation and enhances detection sensitivity. These combined advantages have promoted the widespread application of cellulose-based materials as sustainable and environmentally friendly platforms for electrochemical sensing [10].

Starch is another renewable and biodegradable polysaccharide that has attracted increasing interest as a sustainable sensing material because of its low cost, natural abundance, biocompatibility, and film-forming properties. Composed of amylose and amylopectin, starch provides numerous hydroxyl groups that facilitate surface modification and composite formation with conductive materials. Although its intrinsic electrical conductivity is limited, starch-based composites have demonstrated promising performance in disposable and low-cost electrochemical sensors for environmental, food, and biomedical applications [11].

Thus, recent research has included the design of eco-friendly electrochemical sensors using biodegradable polymeric materials. These techniques are implemented to minimize the environmental impact of analytical devices and at the same time achieve or even enhance the sensing efficiency of the system. Advantages of biodegradable polymers are biocompatibility, renewability, low toxicity, and the capability to produce stable films on electrode surfaces. Moreover, their functional groups support effective immobilization of biomolecules and facilitate efficient electron transfer processes. These traits are a draw toward the next-generation sensing platforms and will potentially make biodegradable polymers the cornerstone of the development of green analytical technologies and eco-friendly electrochemical sensors.

## 2. Green Electrochemical Sensors

Green electrochemical sensors in development are based on the principles of green chemistry, as well as green analytical chemistry. Green chemistry is an approach which involves the reduction or outright removal of the use and formation of harmful chemical substances in the design and production of chemical products. In particular, this focus is on minimizing the environmental impact of reagents, solvents, and materials used in analytical chemistry activities [12]. Electrochemical sensors are considered compatible technologies with green analytical techniques, as they are capable of direct sample analysis and frequently do not require additional chemical reagents. Due to real-time and on-site testing, these sensors can curb the generation or utilization of hazardous chemicals. Their small sample volume leads to substantial waste generation reduction [13].

In this context, sensor design in accordance with green chemistry principles includes elements such as the use of environmentally friendly solvents, the preference for non-toxic electrode materials, and the application of energy-efficient production methods. In particular, environmentally friendly solvents such as deep eutectic solvents (DESs) and ionic liquids are increasingly used in the development of electrochemical sensors [12].

**Figure 1:**
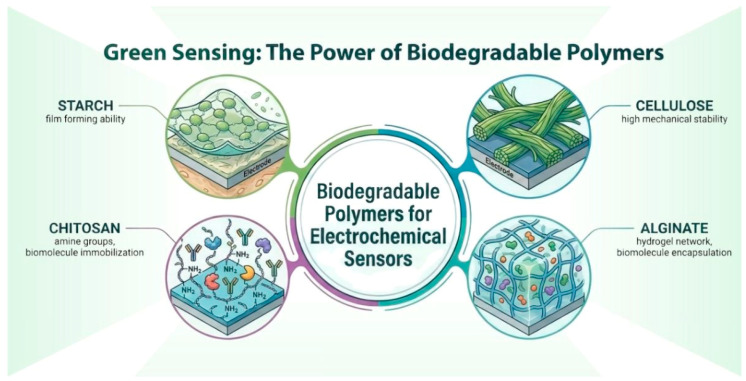
Schematic illustration of biodegradable polymers commonly used in electrochemical sensor fabrication. Natural polymers such as chitosan, starch, alginate, and cellulose provide advantageous properties including film-forming ability, mechanical stability, hydrogel network formation, and functional groups for biomolecule immobilization, making them suitable for electrode surface modification.

Sustainability is an important consideration in green electrochemical sensor design. The objective is to obtain technology by adopting biodegradable materials in the manufacturing process and to minimize the use of toxic chemicals as much as possible. Furthermore, low energy consumption and minimal waste generation during the production of sensors are also fundamental design criteria. Natural polymers, including chitosan, starch, alginate, and cellulose, are among the most commonly used materials for electrode surface modification due to their biodegradability, non-toxicity, and good film-forming properties, as presented in Figure 1. These biopolymers act as stabilizers and/or binding agents in nanoparticle synthesis for controlling the size and shape of the nanoparticles and for improving the biocompatibility of the sensors [14].

The preference for these natural polymers is largely associated with their chemical structures and functional groups. Examples include chitosan contains amino (–NH_2_) and hydroxyl (–OH) groups [15], which enable strong interactions with metal nanoparticles and facilitate the formation of stable films on electrode surfaces. Cellulose, with its linear structure composed of β-1,4-glycosidic linkages and abundant hydroxyl groups [16], provides high mechanical strength and hydrophilicity. Alginate possesses carboxylate (–COO^−^) groups that allow for ionic crosslinking and gel formation, enhancing structural stability [17]. Starch, consisting of amylose and amylopectin, offers good film-forming ability and biocompatibility [18]. These structural characteristics contribute significantly to improving the stability, sensitivity, and overall analytical performance of electrochemical sensors.

### 2.1. Environmentally Friendly Electrochemical Sensor Design Criteria

Environmentally friendly electrochemical sensor design attempts to minimize environmental consequences whilst still ensuring high performance in terms of sensitivity, selectivity, and stability. The incorporation of green chemistry in sensor development is now essential, as highlighted in recent years by the growing concern about toxic materials, hazardous solvents, and non-biodegradable material in the sensor production process. In this context, the development of green sensors aligns with the United Nations Sustainable Development Goals (UN SDGs), particularly those related to responsible consumption and production (SDG 12), climate action (SDG 13), and good health and well-being (SDG 3) [19]. Moreover, the application of the twelve principles of green chemistry, such as the use of safer solvents and renewable materials, waste prevention, energy efficiency, and the design of less hazardous chemical syntheses [20], plays a critical role in guiding the fabrication of sustainable electrochemical sensors. By incorporating these principles, researchers aim to reduce the ecological footprint of sensor production while ensuring high performance and long-term environmental compatibility and life cycle considerations.

The integration of renewable and biodegradable materials has become a central strategy in the development of sustainable electrochemical sensors. Among these materials, cellulose, chitosan, alginate, and starch are widely explored because of their intrinsic biocompatibility, biodegradability, and versatile functional properties. These biopolymers can be employed as sensing matrices or immobilization supports, offering environmentally benign alternatives to traditional synthetic polymers in electrochemical sensing systems [21]. Green synthesis methods also play a crucial role in the development of sustainable electrochemical sensors. Unlike conventional fabrication processes, which often rely on hazardous chemicals and toxic solvents, green synthesis employs environmentally friendly solvents and electropolymerization techniques that minimize waste generation, reduce energy consumption, and lessen the environmental impact of sensor fabrication [22].

**Figure 2:**
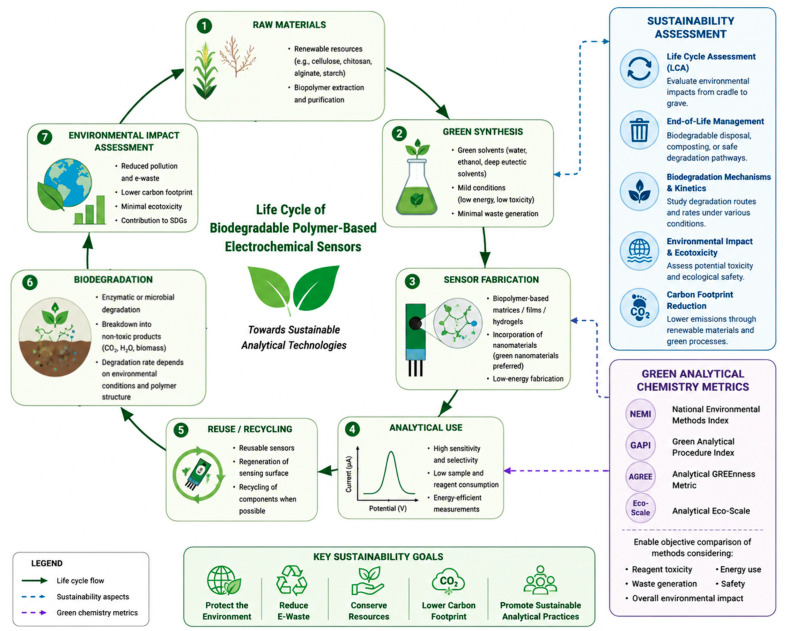
Life cycle and sustainability assessment framework for biodegradable polymer-based electrochemical sensors. The framework highlights the major stages of sensor development, including renewable raw materials, green synthesis, sensor fabrication, analytical application, reuse or recycling, biodegradation, and environmental impact assessment. The figure also summarizes key sustainability evaluation tools, including Life Cycle Assessment (LCA) and green analytical chemistry metrics (NEMI, GAPI, AGREE, and Analytical Eco-Scale), which support the comprehensive environmental assessment of electrochemical sensing platforms.

The overall sustainability framework for biodegradable polymer-based electrochemical sensors is illustrated in Figure 2. Beyond the use of renewable biopolymers, the development of sustainable electrochemical sensors should be evaluated from a holistic perspective that considers the entire life cycle of the device, from raw material selection and green synthesis to sensor fabrication, analytical application, end-of-life management, and environmental fate. In addition to achieving high analytical performance, sustainable sensor design aims to minimize energy consumption, hazardous reagent use, waste generation, and greenhouse gas emissions throughout the manufacturing and operational processes. The biodegradation behavior of polymeric materials, including their degradation mechanisms and kinetics under different environmental conditions, also plays a crucial role in determining the long-term environmental compatibility of these sensing platforms. Furthermore, the incorporation of conductive nanomaterials may influence both degradation pathways and potential ecotoxicological effects, highlighting the importance of balancing analytical performance with environmental safety. Standardized sustainability evaluation tools, such as Life Cycle Assessment (LCA), the National Environmental Methods Index (NEMI), the Green Analytical Procedure Index (GAPI), the Analytical GREEnness Metric (AGREE), and the Analytical Eco-Scale, provide objective and complementary approaches for assessing the environmental performance of electrochemical sensing platforms. The integration of these assessment methodologies enables systematic comparison of sensor designs, facilitates the identification of environmental hotspots, and supports the development of next-generation electrochemical sensors that are not only analytically efficient but also environmentally responsible and fully aligned with the principles of green analytical chemistry and sustainable development [22,23,24].

### 2.2. Comparison of Conventional and Sustainable Electrochemical Sensor Approaches

In most instances, green electrochemical sensors are more design-driven than conventional sensors. Because conventional sensors tend to only be emphasized analytically, sensitive (or accurate, reproducible) green electrochemical sensors are designed so that this performance is kept at bay in a less environmental way. In particular, toxic solvents and energy-intensive methods for their construction is much mor significant with conventional sensors; green sensors are made based on more sustainable materials and therefore result in lower energy consumption [25]. In this way, such sensors are more analytical and environmentally friendly technological approaches [26].

**Figure 3:**
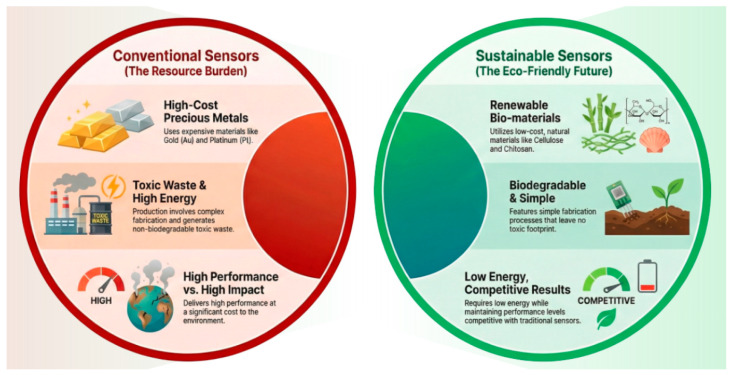
Comparison between conventional sensors and sustainable sensors in terms of material usage, energy consumption, and environmental impact. Sustainable sensors offer eco-friendly advantages through the use of renewable biomaterials, lower energy requirements, and reduced toxic waste generation compared to traditional sensor systems.

From the functional side, both sensor types use electrochemical process, but both are more focused on application and operational advantages compared to other sensor types. Electrochemical sensors already possess rapid, portable, and low-cost tools to do analysis; green electrochemical sensors bring down reagent consumption and waste generation and allow in situ analysis to a minimum [27]. This enables more sustainable and realistic analysis techniques, particularly for environmental monitoring and food safety in food production [12]. As illustrated in Figure 3, a comparative overview of these sensor types highlights their respective advantages and contributions.

From a materials and fabrication perspective, the advantages of green sensors become even more evident. In conventional sensors, performance enhancement is often achieved using toxic or environmentally harmful chemicals, whereas green electrochemical sensors rely on biodegradable polymers, naturally derived components, and green synthesis methods [28]. In addition, the environmental impact of these sensors can be quantitatively evaluated using greenness metrics such as NEMI, GAPI, and AGREE [26]. In this respect, green electrochemical sensors go beyond classical sensor technologies, emerging as next-generation systems optimized both analytically and ecologically.

## 3. Biodegradable Polymers

Recent years have seen growing concern over the environmental impact of petroleum-based polymers and other non-biodegradable materials commonly used in the fabrication of electrochemical sensors. Their persistence in the environment and contribution to pollution have encouraged researchers to explore more sustainable and eco-friendly alternatives. In this context, natural polymers have attracted increasing attention as promising candidates for sensor development. Owing to their biodegradability and biocompatibility, these materials can break down into non-toxic products, making them well suited for applications aligned with the principles of green analytical chemistry. Among the most widely used natural biopolymers are cellulose, chitosan, alginate, and starch.

### 3.1. Cellulose

**Figure 4:**
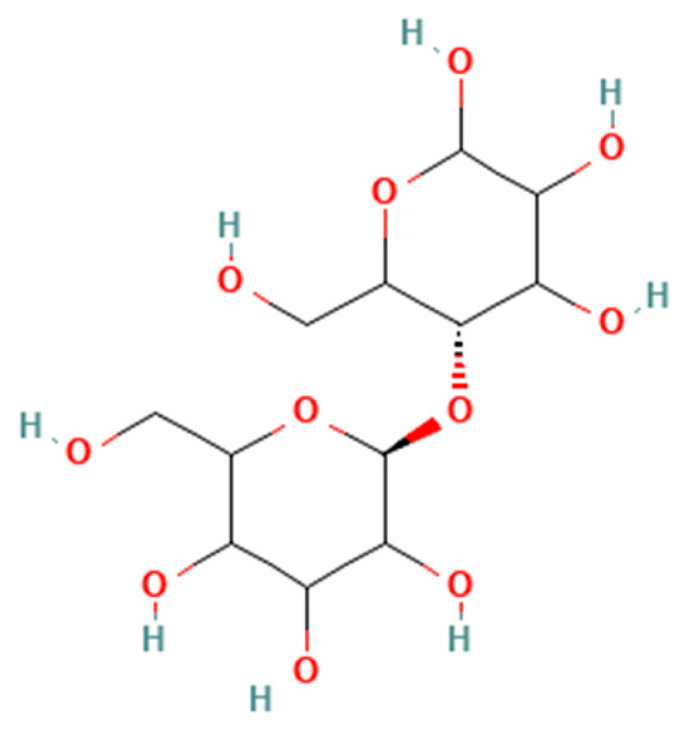
Chemical structure of cellulose.

Cellulose, an important polysaccharide, is the most widely used biopolymer in the world. Cellulose is a homopolymer that contains no sugar units other than glucose [29]. Cellulose is a symmetric biopolymer composed of β-1,4-linked glucan units with the empirical formula (C_6_H_10_O_5_)_n_, and it contains a high density of hydroxyl groups on its surface (Figure 4). It can be derived from various sources, including cotton, wood, algae, and bacteria, and its structure and properties vary depending on its origin. Owing to its renewability, high surface area, and tunable functional groups, cellulose is widely utilized in sensor development. Modification of sensor surfaces with cellulose enhances both selectivity and sensitivity [30]. In recent years, the combination of cellulose with carbon-based nanomaterials has improved its adsorption properties by modifying its surface and has provided enhanced selectivity [31].

Cellulose technology shows superior mechanical properties, renewability, and chemical stability, providing a viable substrate in the electrochemical sensors field for sustainability. Its large number of hydroxyl groups facilitates multiple surface functionalization and can be employed in the use of conductive nanomaterials. Nanocellulose derivatives provide additional increased surface area and enhanced analyte sorption. Cellulose itself is electrically insulating; thus, more conductive materials are generally required for efficient electron transfer. Future research could use this to generate conductive materials such that synthetic conductive additives can be minimized and sustainability maintained [32,33].

### 3.2. Chitosan

**Figure 5:**
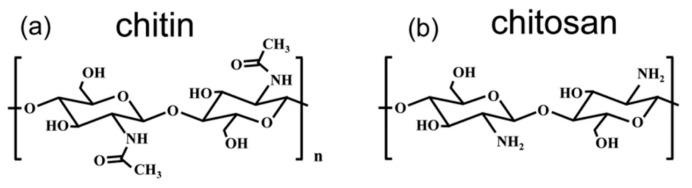
Chemical structure of chitin (**a**) and chitosan (**b**).

Chitosan is a biocompatible and multifunctional material derived from chitin. Chitin is most commonly obtained from the exoskeletons of arthropods or from the cell walls of fungi and yeasts. Chitin has limited applicability due to its insolubility in most conventional solvents. To address this limitation, chitosan is produced through the deacetylation of chitin. Structurally, chitosan is a linear copolymer consisting of β-(1→4)-linked 2-amino-2-deoxy-D-glucopyranose and 2-acetamido-2-deoxy-D-glucopyranose units [6]. Due to its large surface area, renewability, and biocompatibility, it is widely used in sensor development [34]. Furthermore, its ability to easily bond with various nanomaterials, functional groups, and polymers significantly expands its application area (Figure 5). Chitosan plays an important role in enhancing the sensitivity, selectivity, and stability of sensors [2,35].

Compared with the other biodegradable polymers reviewed, chitosan is arguably the most versatile material for electrochemical sensor fabrication because of its abundant amino (–NH_2_) and hydroxyl (–OH) groups, which provide excellent binding sites for enzymes, nanoparticles, and biomolecules. These functional groups facilitate efficient electron transfer and improve the stability of surface modifications. Consequently, chitosan-based sensors generally exhibit high sensitivity and low detection limits across a broad range of analytes. However, the intrinsic electrical conductivity of chitosan is relatively poor, making its combination with conductive nanomaterials such as graphene, carbon nanotubes, or metallic nanoparticles almost essential for high-performance sensing. Furthermore, chitosan films may exhibit limited long-term stability under highly acidic conditions, and variations in the degree of deacetylation may affect reproducibility between studies. Despite these limitations, the ease of processing, excellent biocompatibility, and low environmental impact make chitosan one of the most promising biodegradable polymers for sustainable electrochemical sensors.

### 3.3. Alginate

**Figure 6:**
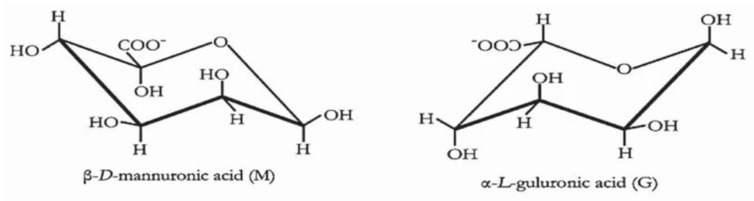
Chemical structure of alginate.

Alginate is a naturally occurring marine biopolymer primarily derived from brown seaweeds such as *Laminaria*, *Microcystis*, and *Ascophyllum*, and can also be biosynthesized by certain bacterial species including *Azotobacter* spp. and *Pseudomonas* spp. In its natural state, alginate is present as salts of divalent and monovalent cations, predominantly calcium, magnesium, and sodium [36]. Structurally, it is composed of a linear block copolymer of β-(1→4)-linked β-D-mannuronic acid (M) and α-(1→4)-linked α-L-guluronic acid (G) residues (Figure 6). The physicochemical properties of alginate vary significantly depending on the mannuronate-to-guluronate (M/G) ratio, with approximately 200 distinct alginate types identified in nature based on this compositional variability [37]. Alginate has attracted considerable attention in sensor modification due to its hydrogel-forming capability, the presence of functional hydroxyl and carboxylate groups in its chemical structure, and its biocompatibility, biodegradability, and high water solubility. These properties not only enhance the sensitivity of the sensor but also enable analytical processes that are consistent with the principles of green chemistry [38].

Alginate provides a number of attractive properties considering its hydrogel-forming capacity and carboxylate groups that facilitate efficient immobilization of biomolecules through ionic crosslinking. The highly hydrated three-dimensional network promotes both analyte diffusion and preservation of biological activity, and hence, is especially desirable for biosensor purposes. However, alginate has poor intrinsic electrical conductivity and relatively weak mechanical strength, especially under prolonged operational conditions. Thus, conductive fillers and reinforcing nanomaterials are frequently used to improve sensor performance. In the future, research will also target improving the electrical conductivity and structural durability of alginate-based sensing platforms while maintaining their biodegradability.

### 3.4. Starch

**Figure 7:**
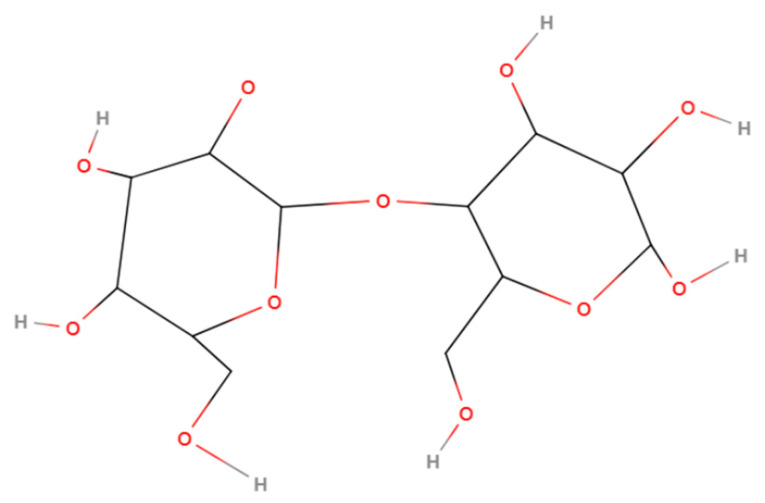
Chemical structure of starch.

Starch is a widely used carbohydrate biopolymer. It possesses gelling, texturizing, and film-forming properties. Due to its film-forming properties, it is used in the development of sensors with modified electrodes [31]. The biodegradable, multifunctional, low cost, and environmentally friendly nature of starch has led to its increased use in sensor applications in recent years [39]. Starch, commonly obtained from potatoes and cassava, is a naturally occurring carbohydrate; therefore, it is abundant in nature, inexpensive, and non-toxic. Furthermore, because it consists of only two types of polysaccharides, it is biodegradable [40]. The hydroxyl groups present in the polysaccharide chains of amylose and amylopectin (Figure 7) confer flexibility, high film-forming capacity, and strong binding ability, making starch a suitable material for the modification and enhancement of electrochemical sensors [41].

Starch is an inexpensive, renewable, and biodegradable polymer with excellent film-forming properties and high availability. It provides environmental compatibility, which makes it one of the most attractive materials for disposable sensing devices. But starch-based sensors generally have limited mechanical stability, poor water resistance, and relatively low electrical conductivity. Most of these limitations can be resolved by blending with other polymers or nanomaterials in order to attain the desired analytical performance. But starch is a good candidate for the development of environmentally benign sensors under these circumstances due to its low cost and wide availability.

Cellulose, chitosan, alginate, and starch share the advantages of biodegradability and renewable origin, yet each material exhibits unique physicochemical features that affect its applicability in electrochemical sensing. Differences in molecular structure, functional group distribution, film-forming ability, mechanical strength, electrical conductivity, and biocompatibility dictate both the fabrication methodology and the sensing performance. A systematic comparison of these materials is presented in Table 1 to facilitate the selection of appropriate biodegradable polymers according to the intended sensing application. More importantly, the comparison reveals that the primary role of each polymer in sensor design is governed by its intrinsic chemical structure and functional groups [42]. Chitosan, containing abundant amino (–NH_2_) and hydroxyl (–OH) groups, primarily functions as an efficient biomolecule immobilization matrix, promoting strong interactions with enzymes, antibodies, nanoparticles, and other recognition elements while facilitating electron transfer. Alginate, characterized by carboxylate (–COO^−^) and hydroxyl (–OH) groups, mainly acts as a hydrogel scaffold, enabling ionic crosslinking, efficient analyte diffusion, and the preservation of biological activity. In contrast, cellulose, composed of β-(1→4)-linked D-glucose units with a high density of hydroxyl (–OH) groups, provides excellent mechanical strength, structural stability, and a versatile platform for surface functionalization. Starch, consisting of amylose and amylopectin chains rich in hydroxyl (–OH) groups, is generally employed as a renewable, low-cost, film-forming substrate for disposable sensing platforms. Beyond their biodegradability and biocompatibility, the mechanical characteristics of biopolymers are critical determinants of their biomedical performance. Optimized mechanical strength, elasticity, and structural stability facilitate their application in regenerative medicine, particularly in bone tissue engineering and wound healing, where temporary support and controlled degradation are essential [43]. Although biodegradable polymers are widely recognized for their excellent biocompatibility and sustainability [4], which make them suitable candidates for biomedical and environmental applications, their broader utilization is constrained by pronounced batch-to-batch variability and limited opportunities to fine-tune their mechanical and electrical performance [44]. Collectively, these observations indicate that biodegradable polymers should be selected not only according to their biodegradability but also according to the specific functional role dictated by their chemical structures, thereby providing a rational framework for the design of sustainable electrochemical sensors.

## 4. Use of Biodegradable Polymers in Sensors

Rapid advances in science and technology, coupled with energy scarcity, increasing heavy metal contamination, and environmental pollution, have intensified the demand for sensitive, selective, and cost-effective detection technologies. In this context, the development of electrochemical sensors has gained significant importance due to their high sensitivity, rapid response, and portability. To further enhance the performance of these sensors, the incorporation of biodegradable polymers and their integration with green chemistry principles has emerged as a prominent research area.

Polymers are essential materials in the fabrication of a wide range of sensors. Sensors produced using biodegradable polymers offer notable advantages, including abundant availability of raw materials, low production costs, biodegradability, flexible design, and high sensing performance, making them a promising option for reducing waste generation. However, a key limitation is the relatively small number of sensors fabricated entirely from biodegradable polymers.

In this study, the use of biodegradable polymers such as cellulose, chitosan, alginate, and starch in sensor development is reviewed, and representative examples from the literature are presented in Table 2.

In general, several interesting trends emerge from the analyses presented in Table 1. Chitosan is the most studied biodegradable polymer, in particular because of its good film-forming ability, high density of amino groups, and compatibility with conductive nanomaterials. Cellulose-based sensor systems are widely used for monitoring of environmental and biomolecule levels because of their mechanical properties and ease of surface modification. Alginate is used most of the time for hydrogel-based biosensors and fluorescence sensing platforms, and starch-based materials are mainly used for disposable and low-cost sensing devices. Differential pulse voltammetry (DPV) is the most commonly used electrochemical method in polymer systems, while square wave voltammetry (SWV) and amperometry are the next most frequently employed for trace level analysis owing to their high sensitivity. Glassy carbon electrodes (GCEs), carbon paste electrodes (CPEs), and screen-printed carbon electrodes (SPCEs) are the most prevalent sensing platforms based on their desirable electrochemical properties and simple-to-modify processes. And increasingly, biodegradable polymers are co-constructed with graphene derivatives, carbon nanotubes, or metallic nanoparticles, to enhance electron transfer and minimize detection limits, indicating the clear trend in hybrid sensing platforms addressing the balance between analytical performance and sustainability.

Across all four biodegradable polymers, several common design strategies can be identified. First, biodegradable polymers primarily serve as matrices for immobilizing conductive nanomaterials and biorecognition elements rather than acting as conductive sensing materials themselves. Second, hybridization with graphene, carbon nanotubes, or metal nanoparticles represents the dominant electrode modification strategy to compensate for the inherently low electrical conductivity of biopolymers. Third, although the sensing mechanisms vary according to the target analyte, improvements in analytical performance are consistently achieved through enhanced electron transfer, increased electroactive surface area, and more efficient biomolecule immobilization. These common trends indicate that sensor performance is governed by similar design principles regardless of the specific biodegradable polymer employed.

Overall, each biodegradable polymer exhibits distinct advantages and limitations depending on the sensing application. Chitosan provides superior biomolecule immobilization owing to its amino-rich structure, whereas cellulose contributes excellent mechanical stability and structural robustness. Alginate is particularly advantageous for hydrogel-based biosensors due to its hydrated three-dimensional network, while starch offers an inexpensive and environmentally sustainable platform for disposable sensors. Nevertheless, none of these polymers simultaneously provide high electrical conductivity, mechanical robustness, and biodegradability. Consequently, recent studies increasingly combine biodegradable polymers with conductive nanomaterials, including graphene derivatives, carbon nanotubes, metal nanoparticles, and conductive polymers, to overcome these limitations. Although these hybrid systems substantially improve analytical performance, they may partially reduce the overall sustainability of the sensor because some nanomaterials are not biodegradable. Therefore, future research should focus on balancing analytical performance with environmental sustainability through the development of fully biodegradable conductive materials and standardized sustainability assessment protocols [45].

**Table 2:** Biodegradable polymers applications.

Biodegradable Polymer	Analyte	Sensor	Detection Method	LOD	Sample	Ref.
Chitosan	Paracetamol	MWCNTs/CTS–Cu	DPV	0.024 μmol L^−1^	Tablet and human serum	[46]
Chitosan	Paracetamol	CS–CPE	SWV	5.08 × 10^−7^ mol L^−1^	Natural waters, tablets, and urine	[47]
Chitosan	Mesalazine Folic acid	CNT-NH_2_/chitosan/GCE	SWV	3.1 × 10^−9^ M	Human serum and pharmaceutical products	[48]
Chitosan	Dopamine Paracetamol	RGO-CB-CTS/GCE	SWV	2.0 × 10^−7^ mol L^−1^ 5.3 × 10^−8^ mol L^−1^	Urine	[49]
Chitosan	Rabeprazole	N-CNTs-CHIT/GCE	Amperometrik	60.0 ng mL^−1^	Pharmaceutical tablets	[14]
Chitosan	4-aminophenol	Graphene–chitosan modified GCE	DPV	0.057 μM	Water and paracetamol tablets	[50]
Chitosan	Epinephrine	GQD–chitosan modified CPE	Amperometrik SWV	0.3 nM	Blood serum and injection solution	[51]
Chitosan	Erythromycin Azithromycin Clarithromycin Roxithromycin	BIA-AMP modified SPCB	Amperometrik	0.191 µmol L^−1^ 0.153 µmol L^−1^ 0.161 µmol L^−1^ 0.186 µmol L^−1^	Water and pharmaceutical	[52]
Chitosan	Mesalazine and folic acid	CNT-NH_2_/chitosan/GCE	SWV	3.1 × 10^−9^ M	Human serum and pharmaceutical dosage form	[48]
Chitosan	Sulfamethazine	M-Chs-Aci/CPE	DPV	0.021 μM	Milk	[53]
Chitosan	Thiamethoxam pesticide	ZnO/Bi_2_O_3_/Bi_2_S_3_/MIP/FTO	Photoelectrochemical	3.32 × 10^−13^ mol/L	Water and soil	[54]
Chitosan	Dopamine	CS/PBNPs	Colorimetric	0.55 μM	Human serum	[9]
Chitosan	Hydrogen peroxide	CS/PVA-Cu-TPA	Fluorescence	0.1 μM	Milk	[55]
Chitosan	Phenolic compounds	Gr-Au-Chit/Tyr	DPV	0.016 μM	Water	[56]
Chitosan	Opotecan	MIP-Au-CH@MOF-5/GCE	DPV	0.298 nM	Human plasma and nasal	[57]
Chitosan	Bisphenol A	CS–Fe_3_O_4_/GCE	DPV	8.0 × 10^−9^ mol dm^−3^	Plastic	[58]
Chitosan	*o*- and *p*-nitrophenols	RGO-CD-CS	DPV	0.018 μM 0.016 μM	Aqueous	[59]
Chitosan	Ni(II) As(III) Pb(II)	α-Fe_3_O_4_/CS	LSV	3.5 × 10^−9^ mol L^−1^ 3.0 × 10^−6^ mol L^−1^ 1.0 × 10^−4^ mol L^−1^	Sewage water and human urine	[60]
Alginate	PFAS	SA hydrogel-N,F-CD	Fluorescent	0.001 ppt	Potable water	[61]
Alginate	Heksanal PV	PVA/SAG/S PVA/SAG/C	Colorimetric	-	Soybean and olive oils	[62]
Alginate	HBsAg	AHCS	ELISA	0.24 ng/mL	Serum	[63]
Alginate	Cd(II) Pb(II) Cu(II)	SWCNTs-SA/GCE	DPASV	31 nM 0.1 nM 1 nM	Tap water	[64]
Alginate	L-Tryptophan D-Tryptophan	Trp/SA/CuNPs/rGO/GCE	DPV	0.205 μM 0.319 μM	Food	[13]
Alginate	Lactate Glucose	Alginate/TNT Scaffolds	Colorimetric	0.069 mM 0.044 mM	Cellulose paper	[65]
Alginate	Tetracycline	CDs	Fluorescence	2 μM	Aquatic environment	[66]
Alginate	Nöron spesifik enolaz	Fe^3+^-alginate	SWV	0.447 pg	Human serum	[67]
Alginate	Histamine	CDs@Ni/Alg	Fluorescence	0.63 nM	Blood, urine, fish	[68]
Alginate	Pyrophosphate	PEGDA-CDs@Cu/Alg	Fluorescence	37.24 μM	-	[69]
Cellulose	Cd^2+^ Pb^2+^ Uric acid 17β-estradiol	MNC/SPCEs	DPV	1.01 μM 0.43 μM 1.8 μM 0.58 μM	Artificial sweat	[70]
Cellulose	*Mycobacterium tuberculosis*	NH_2_-rGO/TEMPO-NCC	DPV	3.14 × 10^−14^ M	*Mycobacterium genomic* DNA	[71]
Cellulose	Cholesterol	Si-GO-g-CMNC	DPV	7.4 μmol/L	Clinical blood	[72]
Cellulose	Phenol Catechol o-Cresol 4-Chlorophenol	Tyr/CTAB-NCC/QDs	DPV	0.082 μM 0.125 μM 0.007 μM 0.021 μM	Lake water	[73]
Cellulose	Glucose	TEMPO-CNC Glucose	Chronoamperometry	0.004 mM	Cell Culture	[74]
Cellulose	Glucose	Graphene-nanocellulose paper	Amperometry	0.270 ±1 μM	*Escherichia coli* O157:H7	[75]
Cellulose	Glucose	PPy/CNC/GOx	DPV	50 ± 10 µM	-	[76]
Cellulose	Hg(II)	PA6/CNW:rGO	DPV	0.52 μM	-	[77]
Cellulose	Atrazine	CLs/PGE	SWV	0.008 ng/mL	Drinking water	[78]
Cellulose	Xanthine	Nanocellulose/xanthine oxidase/GCE	DPV	7.96 nM	Fish	[79]
Cellulose	Tetracycline	SWCNT/TOCNF-PEI	DPV	0.180 µmol L^−1^ 0.112 µmol L^−1^	Effluent	[80]
Cellulose	Lactate	PVA/CNCs@PDA-AuNPs	DPV	0.31 mM	Human Sweat	[81]
Cellulose	Catechol Hydroquinone	CLC/GCE	Amperometry	0.4 μmol/L 0.47 μmol/L	Domestic lake water	[82]
Cellulose	Sulfamethoxazole	CS-AgNPs	SWV	0.04 µM	Meat	[83]
Cellulose	Glucose	-	CV	-	Rat interstitial fluid	[84]
Potato Starch	Hg(II)	MC/GCE	SWASV	-	River water	[85]
Potato Starch	Estriol	RGO-GNPs-PS/GCE	LSV	0.48 μmol L^−1^	Water and urine	[86]
Potato Starch	Tetracycline	CB-PS/GCE	DPV	1.15 μmol L^−1^	Water and milk	[87]
Starch	Cholesterol	PANI/MWCNTs/Starch-CPE	CV	0.01 mM	Cow milk	[88]
Potato Starch	Catechol	Tyr-ND-PS/GC	DPV	3.9 × 10^−6^ mol L^−1^	River and tap water	[89]
Starch	Epinephrine	EP-MIP	DPV EQCM	40 ppb 290 ppb	Blood plasma	[90]
Starch	Hydrogen peroxide	PCS-HB-CPE	CV	0.032 mM	Glucose	[91]
Starch	Caffeine	CuS NPs MCPE	DPV	18 × 10^−9^ M	Food	[92]
Starch	Caffeine	ZnO NPs MGCE	DPV	0.038 μM	Tea and coffee	[93]
Starch	Transferrin	MIP film-coated EQCM	DPV	20 ppb	Blood plasma	[94]
Starch	L-Tyrosine D-Tyrosine	SS-CS/GCE	SWV	0.35 0.42	-	[95]
Starch	Folic acid	*γ*-Fe_2_O_3_	DPSV	2.8 nM 48 nM	Pharmaceuticals	[96]
Manioc starch	Dopamine Catechol	RGO-MS/GCE	CV	0.07 μmol L^−1^ 0.04 μmol L^−1^	Water and synthetic urine	[97]
Cassava starch	Acetaminophen Caffeine	GCE-M221-Fe_3_O_4_	DPV	16 µM 23 µM	Headache medicines	[98]
Manioc starch	Herbicide diquat	ND-MS/GCE	SWV	1.1 × 10^−7^ mol L^−1^	Environmental	[99]
Tapioca starch	17-β estradiol	N-TiO_2_-TP/GCE	LSASV	1.7 × 10^−7^ mol L^−1^	Tap water and synthetic urine	[100]
Manioc starch	Tetracycline	ND-MS/GCE	DPV	2.0 × 10^−6^ mol L^−1^	Water	[101]
Sago starch	Japanese encephalitis virus	CNPs-SPCE	EIS	2 ng·mL^−1^	Human serum	[102]
AHS starch	Dopamine	NPC-GCE	DPV	2.74 nM	Blood serum and urine	[103]
Cellulose and starch	Paracetamol	CPE/S/NanoCo	DPV	9.9 × 10^−10^ M	Tablets	[104]

CTS: chitosan; CS: Chitosan; MWCNTs: multi-walled carbon nanotubes; CD: cyclodextrin; RGO: reduced graphene oxide; SPCB: super P carbon black film; LSV: linear sweep voltammetry; PFAS: per- and polyfluoroalkyl substance; SA: sodium alginate; AHCS: an alginate hydrogel-embedded capillary sensor; HBsAg: hepatitis B virus surface antigen; ELISA: enzyme-linked immunosorbent assay; DPASV: differential pulse anodic stripping voltammetry; CDs: carbon dots; MNC: microbial nanocellulose; SPCEs: screen-printed carbon electrodes; NH_2_-rGO/TEMPO-NCC: reduced graphene oxide/2;2;6;6-tetramethylpiperidin-1-yl)oxyl nanocrystalline cellulose; Si-GO-g-CMNC: silylated graphene oxide-grafted-chemically modified nanocellulose; Tyr: tyrosinase enzyme; CTAB: cetyltriammonium bromide; NCC: nanocrystalline cellulose; QDs: quantum dots; TEMPO: 2;2;6;6-tetramethylpiperidine-1-oxyl; CNC: cellulose nanocrystals; PPy/CNC: polypyrrole/cellulose nanocrystal; GOx: glucose oxidase; rGO: reduced graphene oxide; CNW: cellulose nanowhiskers; PA6: polyamide 6; CLs: cellulose; PGE: pencil graphite electrode; SWCNTs: single-walled carbon nanotube networks; TOCNF-PEI: TEMPO-oxidized cellulose nanofiber-polyethyleneimine hybrids; PDA: polydopamine; PVA: poly vinyl alcohol; SWASV: square wave anodic stripping voltammetry; GNPs: gold nanoparticles; PS: potato starch; CB: carbon black nanoballs; EQCM: electrochemical quartz crystal microbalance; CPE: carbon paste electrode; C: cellulose; NanoCo: cobalt nanoparticles; PCS: polyaniline/multi-wall carbon nanotubes/starch; HB: hemoglobin; SS: soluble starch; CS: chitosan; DPSV: differential pulse stripping voltammetry; γ-Fe_2_O_3_: maghemite; MS: manioc starch; M221: mixture ratio of 2:2:1; ND: nanodiamonds; LSASV: linear sweep adsorptive stripping voltammetry; N-TiO_2_: nitrogen-doped titanium dioxide; TP: tapioca; EIS: electrochemical impedance spectroscopy; NPC: nitrogen-inherited porous carbon; AHS starch: the starch of Artocarpus heterophyllus seeds.

A review of the literature shows that chitosan-based electrochemical sensors have been widely studied using a range of analytical techniques, including DPV, SWV, LSV, amperometric, photoelectrochemical, colorimetric, and fluorescence methods. In one study, El Bouabi et al. developed a sensor modified solely with chitosan on a CPE and applied it to the determination of paracetamol in natural water, tablet, and urine samples using SWV. The linearity range was found to be 1.0 × 10^−3^–4.0 × 10^−4^ mol L^−1^ and 2.0 × 10^−4^–8.0 × 10^−7^ mol L^−1^, while the recovery values were found to be in river water (98.00–99.75%) and seawater (99.00–99.25%), respectively [47]. In addition, Mao et al. developed a modified glassy carbon electrode (GCE) incorporating chitosan and multi-walled carbon nanotubes (MWCNTs), which was successfully applied to the determination of paracetamol in tablet formulations and human serum samples using DPV. The sensor exhibited a linear response over the concentration range of 0.1–200 μmol L^−1^, with a sensitivity of 0.603 A mol^−1^ L [46]. Baccarin et al. developed a modified sensor on a GCE using chitosan in combination with RGO and CB for the simultaneous determination of paracetamol and dopamine, and successfully applied it to urine samples using the SWV technique. The linearity range is 3.2 × 10^−6^ to 3.2 × 10^−5^ mol L^−1^ for dopamine and 2.8 × 10^−6^ to 1.9 × 10^−5^ mol L^−1^ for paracetamol. The recovery values are in the ranges of 85% to 101% and 111% to 100% for dopamine and paracetamol, respectively [49]. Similarly, Abd-Elsabour et al. reported the determination of sulfamethazine in milk by employing a magnetic chitosan acetylindole nanocomposite as a modifier on a CPE, with measurements carried out using DPV. The linearity range, repeatability, and stability were found to be 0.08–6.0 μM, 3.83%, and 94.87%, respectively [53]. Li et al. developed a modified GCE incorporating RGO, CDs, and chitosan, and successfully applied it to the simultaneous determination of o- and p-nitrophenols in aqueous solutions using the DPV technique. The linear ranges for o-nitrophenol and p-nitrophenol were 0.12–0.28 μM and 5–40 μM, and 0.06–0.16 μM and 5–40 μM, respectively. The recovery values obtained from river water samples ranged between 98.30% and 100.59% [59]. Ahmed and Fekry developed an electrochemical sensor based on superparamagnetic iron oxide nanoparticles (α-Fe_3_O_4_) embedded in a chitosan film and coated onto a platinum electrode. This sensor was successfully applied to the determination of Ni(II), As(III), and Pb(II) in sewage water and human urine samples using the LSV technique. The calibration curve was constructed only for Ni(II), and the linear range was determined to be 3.0 × 10^−6^ to 1.0 × 10^−4^ mol L^−1^. Additionally, the recovery values for spiked Ni(II), As(III), and Pb(II) in sewage water and human urine samples ranged from 90% to 98%, 92% to 99%, and 90% to 99%, respectively [60].

Previous studies have demonstrated that alginate-based electrochemical sensors have been evaluated using various analytical techniques, such as fluorescence, colorimetric assays, ELISA, DPASV, DPV, and SWV. Chrouda developed a GCE incorporating SWCNTs and alginate, and successfully applied it to the determination of Cd(II), Pb(II), and Cu(II) in tap water using the DPASV technique. The linear ranges for Pb^2+^, Cd^2+^, and Cu^2+^ were found to vary between 10^−9^ and 10^−5^ M. The sensitivities were determined as 3.4 μA μM^−1^ for Pb^2+^, 28.9 μA μM^−1^ for Cd^2+^, and 16 μA μM^−1^ for Cu^2+^ [64]. Wang et al. developed a chiral-selective modified GCE using alginate and copper nanoparticles as chiral recognition materials, and applied it to the determination of L-tryptophan and D-tryptophan enantiomers in food samples using the DPV technique. The linear range for L-tryptophan and D-tryptophan was determined to be 50–500 μM. The recovery values for L-tryptophan in cow milk, goat milk, beef, and millet samples ranged from 90% to 110%, while those for D-tryptophan varied between 95% and 112% [13]. Similarly, Ethesabi et al. constructed a fluorescent sensor by embedding carbon dots into a sodium alginate matrix and successfully applied it to the detection of tetracycline (0–100 μM) in aquatic environments using a fluorescence-based method. The linearity range was found to be between 1 and 20 μM [66].

A number of studies have reported that cellulose-based electrochemical sensors have been investigated using various techniques, including CV, DPV, SWV, amperometry, and chronoamperometry. Zaid et al. developed a novel peptide nucleic acid (PNA)-based electrochemical sensor for the detection of positive and negative *Mycobacterium tuberculosis* DNA samples. The sensor was fabricated on a screen-printed electrode (SPE) using reduced graphene oxide and 2,2,6,6-tetramethylpiperidin-1-yl)oxyl nanocrystalline cellulose. The analytical performance of the sensor was evaluated using the DPV technique. The concentration range was found to be 1 × 10^−8^ M to 1 × 10^−13^ M, while the RSD value for reproducibility was given as 4.46% [71]. Tang et al. developed a screen-printed sensor modified with 2,2,6,6-tetramethylpiperidine-1-oxyl oxidized cellulose nanocrystals for the determination of glucose in cell culture samples, and performed the measurements using the chronoamperometric method. The linear range was stated as 0.1–2 mM and sensitivity was 5.7 ± 0.3 µA cm^−2^∙mM^−1^ [74]. Annu et al. performed the analysis of atrazine in drinking water using the SWV method with an electrochemical sensor developed solely based on cellulose employing a PGE. While the concentration range was found to be between 5.0 ng/mL and 320 ng/mL, the recovery from drinking water was found to be between 98.8% and 99.1% [78]. Zhao et al. also performed the simultaneous determination of hydroquinone and catechol in domestic lake water samples using an amperometric method with a sensor they developed on a GCE based solely on cellulose. The linearity ranges for hydroquinone and catechol were 0.5–3000 μmol/L and 1–3000 μmol/L, respectively. Recovery values in analyses performed on lake water samples range from 98.76% to 101.77% [82].

During the literature survey, starch-based sensors have been reported in combination with various instrumental techniques, including SWASV, LCV, DPV, CV, EQCM, SWV, DPSV, and EIS. Gautam et al. developed a sensor for the determination of cholesterol in cow milk using the CV technique, where the sensor was fabricated on a CPE surface modified with starch as the polymer matrix, aniline as the functional monomer, and MWCNT as the nanomaterial. The linearity range was found to be between 0.032 and 5 mM, while the sensitivity was found to be 800 μA mM^−1^ cm^−2^ [88]. Similarly, Ramu et al. presented the simultaneous analysis of acetaminophen and caffeine in headache medications using the DPV method, based on a GCE modified with cassava starch and γ-Fe_2_O_3_ (maghemite) nanoparticles. Two different linearity ranges were given, 0.05 to 1 μM and 1 to 80 μM, respectively [96]. In another study, Fernandes-Junior et al. reported the detection of Japanese encephalitis virus in human serum using the EIS technique, employing a GCE surface modified with manioc starch and nanodiamond. The linearity range and recovery values from water samples were 5.0 × 10^−6^ to 1.8 × 10^−4^ mol L^−1^ and 86 to 112%, respectively [101]. Additionally, Azab developed a sensor for the determination of paracetamol in tablet formulations by modifying the CPE surface with both cellulose and starch in the presence of cobalt nanoparticles, and the analyses were carried out using the DPV technique. Recovery values of 99.4% to 101.8% per tablet were found within a linearity range of 2.0 × 10^−8^ to 1.5 × 10^−4^ mol L^−1^ [104].

## 5. Future Perspectives

Although remarkable progress has been achieved in the development of biodegradable polymer-based electrochemical sensors, several challenges must be addressed before their widespread commercialization and practical implementation. Future research should focus on improving the electrical conductivity, long-term stability, and mechanical robustness of biodegradable sensing platforms while maintaining their environmental compatibility. The integration of biodegradable polymers with advanced nanomaterials, such as graphene derivatives, carbon nanostructures, metal–organic frameworks, and conductive biopolymers, is expected to further enhance sensor sensitivity, selectivity, and operational durability.

The second direction offers significant prospects: the fabrication of totally degradable and disposable, low-waste sensor systems that offer efficient electronics usage through their entire life cycle. It is in this framework that sustainable electrodes, substrates, packaging materials, and power source manufacturing are a significant research aim. In addition, additive manufacturing techniques, such as printed electronics, 3D printing, etc., could potentially benefit the environmentally friendly sensing devices in a cost-effective and scalable way. Wearable, flexible biosensors in the context of biodegradable polymers also appear to be very attractive for use in future healthcare. It could enable the ongoing monitoring of biomarkers in sweat and saliva and of the various other biological matrices with lower environmental footprints after disposal. Moreover, the coupling of biodegradable electrochemical sensors with wireless communications systems, Internet of Things (IoT) networks, and AI-based data processing schemes could provide possibilities for real-time environmental monitoring, food quality characterization, and personalized medicine. Most notably, studies should be conducted that focus more on the comprehensive characterization of sustainability methods through their Life Cycle Assessment (LCA), greenness indicators, and techno-economic analyses. Guidelines for standardized screening of biodegradability, environmental impact, and analytical performance will be vital both for comparing and accelerating the transition from lab-scale demonstrations to commercialized environments for the various sensor platforms.

Future research on biodegradable polymer-based electrochemical sensors should move beyond material substitution and focus on the development of fully integrated green sensing systems. Paper-based electrochemical devices are particularly promising because paper is low-cost, widely available, mechanically robust, porous, foldable, and compatible with pump-free microfluidic operation; therefore, it can reduce reagent use, sample pretreatment, liquid waste, and overall environmental impact in disposable sensing platforms [105]. In parallel, sustainable printed electrochemical platforms, including screen-printing, inkjet-printing, and 3D-printing, offer scalable routes for producing low-cost and miniaturized sensors using greener materials such as cellulose, polylactic acid, silk proteins, and biochar [106]. These approaches may support the development of transient and disposable electrochemical sensors for environmental, food, clinical, and point-of-care applications. Another important future direction is the integration of biodegradable sensing materials with intelligent and self-powered systems. Recent studies emphasize that green electrochemical sensing systems are increasingly being designed by combining autonomous energy harvesting, biodegradable materials, low-power data transmission, and artificial intelligence-assisted data processing. Self-powered modules such as solar cells, triboelectric nanogenerators, piezoelectric generators, microbial fuel cells, and moisture-enabled electric generators can reduce dependence on external batteries and enable long-term environmental monitoring. Moreover, AI and Edge AI approaches can improve data interpretation, compensate for complex environmental matrix effects, and support real-time decision-making in Internet of Things-based sensing networks [107]. Although biopolymers provide renewability, biocompatibility, biodegradability, and functional groups suitable for sensor modification, their electrical conductivity, mechanical stability, reproducibility, and long-term operational durability still require improvement [42]. In addition, the use of nanomaterials should be carefully optimized because green synthesis routes and renewable precursors are needed to minimize environmental impact while maintaining high analytical performance [108].

Therefore, future studies should focus on establishing standardized biodegradability and Life Cycle Assessment protocols, validating sensor performance under real-world conditions, and integrating biodegradable electrochemical sensors into Internet of Things (IoT) ecosystems. Such integration would enable continuous, real-time, and remote monitoring through wireless communication, cloud-based data management, and edge computing, thereby supporting intelligent environmental surveillance, personalized healthcare, precision agriculture, and smart food quality monitoring. Furthermore, addressing regulatory, manufacturing, storage, and commercialization challenges will be essential to facilitate the large-scale deployment and widespread adoption of biodegradable electrochemical sensing technologies.

## 6. Conclusions

Taken together, cellulose, chitosan, alginate, and starch have established themselves as key biodegradable materials for the fabrication of sustainable electrochemical sensors. Their renewable origin, biodegradability, intrinsic biocompatibility, economic feasibility, and processing versatility provide a solid basis for the development of high-performance sensing platforms with reduced environmental impact. Their diverse physicochemical properties enable them to function as immobilization matrices, film-forming materials, hydrogel scaffolds, or structural supports, providing remarkable flexibility in sensor design. Furthermore, their integration with conductive nanomaterials, including graphene derivatives, carbon nanotubes, metallic nanoparticles, and conductive polymers, has significantly enhanced electron transfer, sensitivity, selectivity, and long-term stability, allowing environmentally friendly sensing platforms to achieve analytical performances comparable to those of conventional sensors. Carbon-based electrodes, particularly glassy carbon, screen-printed carbon, and carbon paste electrodes, remain the predominant substrates because of their excellent electrochemical properties and compatibility with biodegradable polymer coatings. Moreover, a broad range of electrochemical and optical techniques, including CV, DPV, SWV, LSV, EIS, amperometry, colorimetry, and fluorescence methods, have been successfully combined with these biodegradable materials for the determination of pharmaceuticals, biomolecules, pathogens, pesticides, heavy metals, hormones, and environmental pollutants in complex biological, food, and environmental matrices.

After the above-mentioned significant progress, there are still several key issues to solve before biodegradable polymer-based electrochemical sensors can have widespread practical application. Most of the biodegradable polymers inherently exhibit low electrical conductivity, necessitating conductive nanomaterials or hybrid architectures to maintain excellent analytical performance. While these changes significantly enhance sensor sensitivity, they can compromise the overall environmental sustainability of the sensing platform, specifically since several conductive nanomaterials turn out to lack biodegradability. The present literature also inadequately addresses long-term stability, storage conditions, large-scale manufacture, reproducibility, biodegradation behavior, and regulatory approval. It is therefore necessary for future research to develop fully biodegradable conductive materials, optimize green synthesis strategies, and establish standardized sustainability evaluation protocols via Life Cycle Assessment (LCA) and green analytical chemistry metrics, such as NEMI, GAPI, AGREE, and the Analytical Eco-Scale. Over and above material innovation, future biodegradable electrochemical sensors are expected to progress towards an integrated, intelligence-led and application-centric approach. The integration of the biodegradable sensing materials with flexible electronics, wearables, Internet of Things (IoT) devices, wireless data transmission, and artificial intelligence-based data processing may present a great potential for real-time and decentralized monitoring in industries such as healthcare, environmental surveillance, food safety, and precision agriculture. The combined development of these advanced technologies implies that biodegradable polymer electrochemical sensors have gradually become a key component of next-generation sustainable analytical technologies by achieving high analytical performance with low environmental impact and contribute to the wider goals of green chemistry and sustainable development.

## Figures and Tables

**Table 1:** Comparison of cellulose, chitosan, alginate, and starch with respect to their physicochemical properties, sensor fabrication compatibility, applications, and limitations.

Property	Cellulose	Chitosan	Alginate	Starch
Functional groups	–OH	–NH_2_, –OH	–COO^−^, –OH	–OH
Film-forming ability	Excellent	Excellent	Good	Good
Mechanical strength	Excellent	Moderate	Moderate	Low
Biocompatibility	Excellent	Excellent	Excellent	Good
Electrical conductivity	Very low	Low	Very low	Very low
Sensor fabrication compatibility	Excellent	Excellent	Excellent	Good
Typical applications	Heavy metals, glucose, antibiotics	Pharmaceuticals, biomolecules, heavy metals	Biosensors, PFAS, food analysis	Disposable sensors, food analysis
Major limitations	Poor conductivity	Acid stability, poor conductivity	Weak mechanical stability	Low conductivity, water sensitivity

## Data Availability

No new data were created or analyzed in this study. Data sharing is not applicable to this article.
